# Characterizing Forest Change Using Community-Based Monitoring Data and Landsat Time Series

**DOI:** 10.1371/journal.pone.0147121

**Published:** 2016-03-28

**Authors:** Ben DeVries, Arun Kumar Pratihast, Jan Verbesselt, Lammert Kooistra, Martin Herold

**Affiliations:** Laboratory of Geo-Information Science and Remote Sensing, Wageningen University, Wageningen, The Netherlands; University of Maryland at College Park, UNITED STATES

## Abstract

Increasing awareness of the issue of deforestation and degradation in the tropics has resulted in efforts to monitor forest resources in tropical countries. Advances in satellite-based remote sensing and ground-based technologies have allowed for monitoring of forests with high spatial, temporal and thematic detail. Despite these advances, there is a need to engage communities in monitoring activities and include these stakeholders in national forest monitoring systems. In this study, we analyzed activity data (deforestation and forest degradation) collected by local forest experts over a 3-year period in an Afro-montane forest area in southwestern Ethiopia and corresponding Landsat Time Series (LTS). Local expert data included forest change attributes, geo-location and photo evidence recorded using mobile phones with integrated GPS and photo capabilities. We also assembled LTS using all available data from all spectral bands and a suite of additional indices and temporal metrics based on time series trajectory analysis. We predicted deforestation, degradation or stable forests using random forest models trained with data from local experts and LTS spectral-temporal metrics as model covariates. Resulting models predicted deforestation and degradation with an out of bag (OOB) error estimate of 29% overall, and 26% and 31% for the deforestation and degradation classes, respectively. By dividing the local expert data into training and operational phases corresponding to local monitoring activities, we found that forest change models improved as more local expert data were used. Finally, we produced maps of deforestation and degradation using the most important spectral bands. The results in this study represent some of the first to combine local expert based forest change data and dense LTS, demonstrating the complementary value of both continuous data streams. Our results underpin the utility of both datasets and provide a useful foundation for integrated forest monitoring systems relying on data streams from diverse sources.

## Introduction

Recent years have seen a dramatic increase in the attention being given to the plight of tropical forests. This attention is due to the importance that these ecosystems have with regards to global climate change [1, 2], biodiversity loss [3, 4] and ecosystem services [5]. In recognition of the considerable impact human activities are having on tropical forest systems worldwide, a range of initiatives have been launched to mitigate against the adverse effects of tropical forest loss. One such programme—Reducing Emissions from Deforestation and Degradation (REDD+)—is designed to provide incentives to developing countries to reduce deforestation and forest degradation rates and strengthen conservation measurements [6, 7].

Countries wishing to engage in REDD+ are required to undertake monitoring measures enshrined in the Measuring, Reporting and Verification (MRV) framework [7, 8]. Estimates of forest area changes in baseline and reporting periods—termed “Activity Data”—comprise an important component of REDD+ MRV [9]. While the choice of methods and technologies used to fulfill MRV requirements are left up to individual participant countries, satellite remote sensing data have been widely recognized as essential data sources for comprehensive mapping and quantification of forest area change [10]. In addition to the various change detection approaches already existing [11], MRV-related capacity gaps among participant countries [12] have resulted in a surge of new forest change monitoring methods and case studies in the tropics. While deforestation monitoring is operational in many cases, forest degradation is still poorly understood in many areas of the tropics [13–15]. This gap is due to the nature of degradation processes, including complex governance structures and drivers, as well as technical challenges related to degradation monitoring, and thus remains a bottleneck to the implementation of effective MRV systems [16].

Recent years have seen a paradigm shift in satellite-based forest monitoring, with dense time series increasingly being used in favour of conventional bi-temporal image comparison approaches [17]. This shift is largely due to open data policies, such as the decision to release the entire Landsat archive to the public in 2008, which has spurred considerable development in Landsat time series (LTS) based monitoring methods [18]. These methods have allowed for forest change monitoring with gains in both resolution and accuracy in the temporal domain [13, 19–21]. Furthermore, a number of operational forest monitoring systems based on satellite time series have emerged in the tropics, such as the PRODES and DETER systems of the Brazilian Space Agency [22, 23], the Monitoring of the Andean Amazon Project (MAAP) [24], the Global Forest Watch [25, 26] and others.

Not only do forest monitoring methods based on LTS allow for rapid detection of forest disturbance, but they also allow for descriptions of forest change trajectories well beyond what is possible with conventional methods [27, 28]. Change trajectory analysis usually involves the segmentation and/or reduction of a time series to describe the change history at a particular location. Several segmentation methods have been described in the literature. The Breaks For Additive Season and Trend (BFAST) method detects abrupt and gradual changes in time series decomposed into season, trend and noise components [29]. The Detecting Breakpoints and Estimating Segments in Trend (DBEST) method similarly segments time series to measure timing, type and magnitude of changes [30], but without considerations for seasonal variations as in BFAST. The Landsat-based detection of Trends in Disturbance and Recovery (LandTrendR) method segments annual or composited Landsat time series using a series of parameters describing segment length, inter-segment angle and other characteristics of time series trajectories [27]. While these and other time series segmentation algorithms have been proven to be useful in describing changes or other state variables using satellite time series, they have all been developed for regularly timed observations such as MODIS 16-day composites [29], AVHRR GIMMS3g data [30] or annual Landsat composites [27]. Few studies have applied analogous techniques to time series with missing data (“irregular” time series) such as LTS data using all available observations [31].

Even with increasingly sophisticated tools for quantifying and describing forest changes using satellite image time series, the involvement of local people in monitoring activities (such as in Community-Based Forestry projects) is necessary to ensure sustainability [32, 33] and equity [34] in forest management programmes such as REDD+. Community involvement in monitoring activities has also been shown to reduce overall monitoring costs with negligible trade-offs in data quality for certain monitoring applications [35]. Use of community-based monitoring (CBM) data or volunteered geo-information (VGI) data have been previously shown to be promising in such applications as land cover validation [36], climate change impact studies [37] or forest carbon stock estimation [35, 38]. Emerging technologies such as smart phones [35, 39] improve the quality and consistency of these data through functionalities such as integrated photos and geo-tagging capabilities [40].

Another area where CBM or VGI data could add considerable value is in the training and validation of forest change detection methods, since the validation of historical change estimates is often severely limited by a lack of reliable historical reference data [41]. However, very few studies have been undertaken to demonstrate the utility of local monitoring data in such a context. Pratihast et al. (2014) [42] showed that local forestry experts in southern Ethiopia can describe forest changes with much higher thematic details than is possible with satellite time series, but some trade-offs were encountered with regards to spatial coverage and temporal accuracy. Notably, this study found that local experts were particularly adept at describing locations and drivers of low-level degradation [42], a great deal of which is not adequately captured by satellite-based methods [13]. There is currently a need for more research on approaches to integrate CBM or VGI data with satellite time series data to improve the spatial, temporal and thematic quality of forest change estimates.

The objective of this study was to investigate the utility of local expert data combined with LTS-based trajectory analysis to characterize forest change processes. To this end, we investigated three overall research questions:

1. How well can we differentiate between deforestation and forest degradation using local expert data and Landsat time series?
2. What impact does a continuous stream of local expert data have on predictions of forest change types?
3. How can maps of forest change types be used to describe key change processes?

To address these research questions, we used forest disturbance reports collected from 2012 to 2015 by a team of 30 forest rangers in a montane forest area in southwestern Ethiopia and compared them with LTS trajectories. Using all available LTS data, we first derived a series of temporal trajectory metrics from time series of each spectral band and index using an adapted version of the BFAST algorithm [29]. We derived these metrics to describe changes in trend and seasonal amplitudes between time series segments as well as overall time series trend and intercepts. To address the first research question, we combined all local disturbance reports and time series metrics to train random forest models designed to predict deforestation, degradation or stable forest (no change). To address the second research question, we divided the local expert data into training and operational phases and measured the accuracies of predicted models as new training data were added to the models. Finally, to explore the third research question, we used the most important spectral-temporal covariates to map deforested and degraded forests based on LTS as of March 2015.

We studied the relationship between community-based monitoring data and dense LTS over a tropical montane forest system in southern Ethiopia (project setting described below). This work builds upon the work of both DeVries et al. (2015) [13] and Pratihast et al. (2014) [42]. DeVries et al. (2015) mapped annual forest disturbances in this system using dense LTS, for which degradation proved elusive [13]. Pratihast et al. (2014), on the other hand, showed that local rangers in the study area were able to capture degradation sooner than was possible with manual interpretation of very high resolution optical imagery [42]. This study builds upon both of these papers in its attempt to combine community-based monitoring data with dense LTS towards mapping deforestation and low-level degradation with improved confidence and consistency.

## Methods

### Study Area and Project Context

This study was carried out in the UNESCO Kafa Biosphere Reserve (hereafter referred to as “Kafa BR”) in southwestern Ethiopia. The Kafa BR comprises an Afro-montane forest system consisting mostly of highly fragmented moist evergreen forests, forest-cropland matrix landscapes, coffee forests, tree plantations and wetlands. A detailed description of the study area as well as the drivers of deforestation and forest degradation is provided in DeVries et al. (2015) [13].

**Fig 1:**
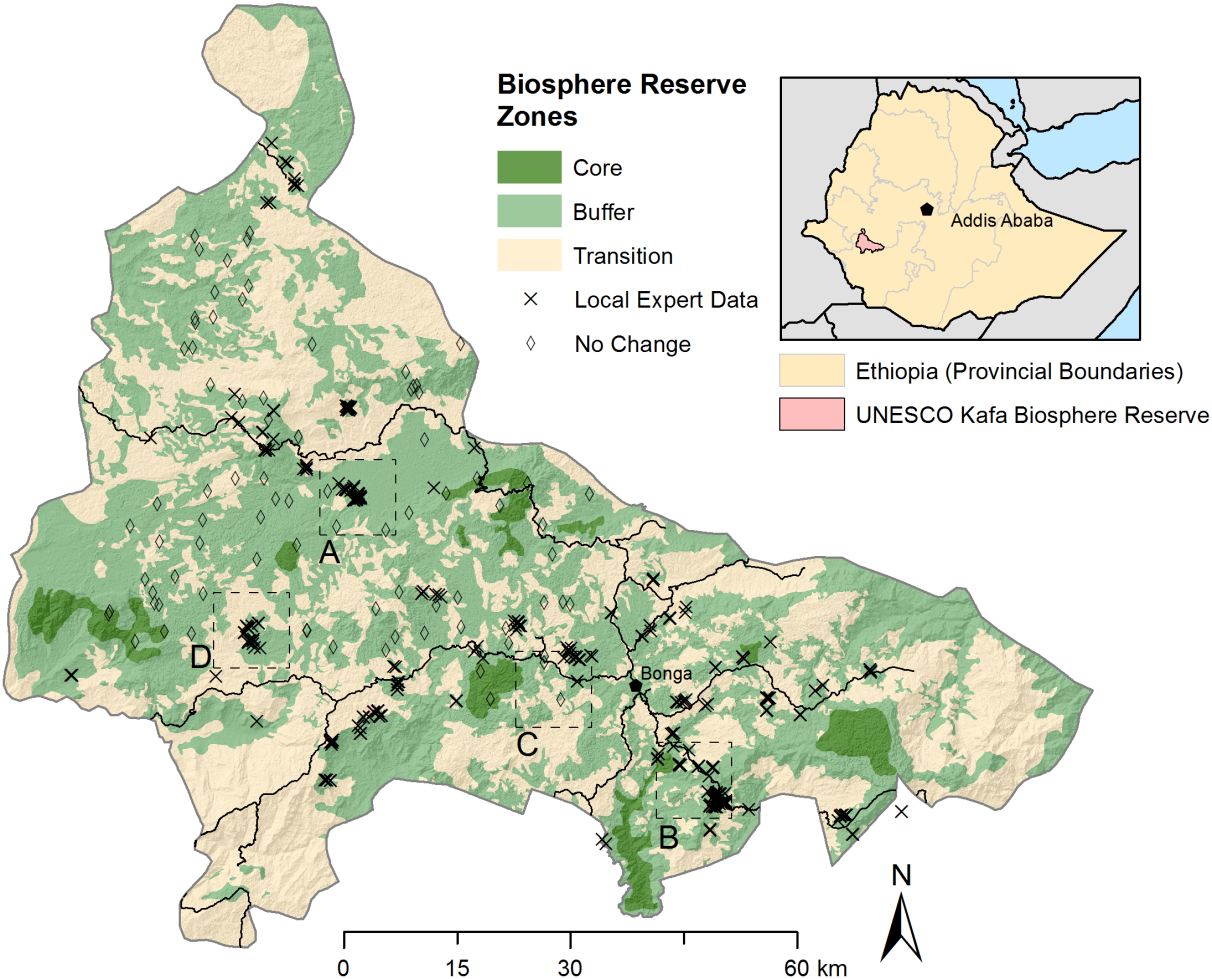
Study area located in the UNESCO Kafa Biosphere Reserve in the Southern Nations, Nationalities and Peoples Republic (SNNPR) state of southwestern Ethiopia. Biosphere Reserve zones and location of local expert disturbance reports (deforestation and degradation) and additional reference data (no-change) are shown. The locations of map tiles from Fig 9 are shown as boxes labeled A to D.

The research in this study was carried out in the frame of a project implemented by the German Nature and Biodiveristy Conservation Union (NABU), in partnership with the Kafa Zone Bureau of Agriculture, the zonal office of the Ethiopian Ministry of Agriculture. This project aimed to reduce carbon emissions from deforestation and forest degradation in the Kafa BR and to promote conservation and sustainable management of remaining forest resources in the area. In line with the projects goals, the region was inaugurated as a Biosphere Reserve in 2011 under the UNESCO Man and the Biosphere (MAB) programme and was zoned according to land use (Fig 1).

As part of these initiatives, 30 forest rangers (hereafter referred to as “local experts”) were recruited to implement forest management, monitoring and community outreach activities in each of the 10 local districts (woredas) within the Kafa BR. As part of their monitoring mandate, local experts were trained in methods and tools to report and describe forest changes, including disturbances (deforestation and degradation) and positive changes (afforestation and reforestation). Fig 1 shows the geo-location of the disturbance reports provided by local experts between 2012 and 2015 which were used in this study. The details of these reports are described below, and have also been described in detail in a previous study in the area [42]. The overall goal of the current study was to develop an integrated monitoring system using the knowledge of the local experts in combination with Landsat time series and very high resolution (VHR) time series [42] to track forest change throughout the Kafa BR.

### Definition of Change Classes

In order to address our first research question, a definition of deforestation and forest degradation is needed. This definition can take on several criteria related to area change, canopy cover change, or other dimensions of the change [16, 43–45]. For example, the IPCC defines degradation as changes negatively affecting carbon stocks in forests which remain forests, where a forest is defined based on area, height and canopy cover thresholds [9]. Degradation can thus occur when a forest is completely cleared, but the total area cleared is less than the area threshold (e.g. 0.5 hectares). Degradation can alternatively occur when a larger area of forest experiences negative changes in forest canopy cover, but the canopy fraction still remains above a defined forest threshold (e.g. 20%).

In this study, we limited the definitions of deforestation and degradation to the tree canopy dimension described above. In other words, if the forest canopy was reduced to below our forest definition canopy cover threshold of 20% at the pixel or plot level, we assigned a “deforestation” label, regardless of the total contiguous area cleared. Any negative changes evident that still resulted in a canopy cover of above 20% thus resulted in a label of “degradation”. We neglected the area-based definition in this study for two reasons. First, it was often difficult to determine with certainty the total area affected from local expert disturbance reports, but canopy condition could be verified using plot photos submitted by local experts. Second, we sought to derive relationships between temporal metrics derived from LTS and change classes derived from local expert disturbance data, and spatial context was thus not considered here.

**Fig 2:**
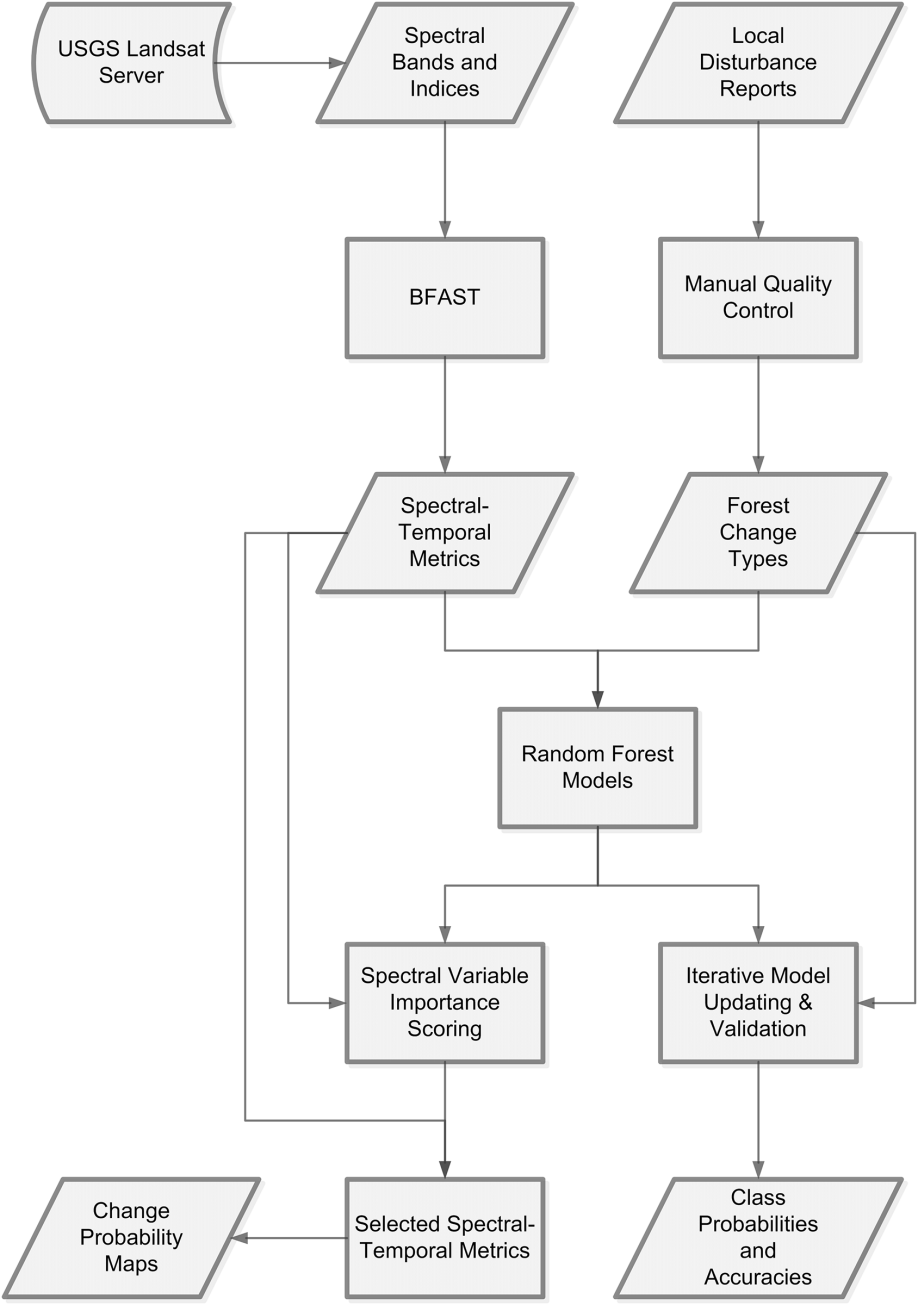
Flowchart of methods used in this study. Processes are shown as rectangles and data and results are shown as parallelograms.

A summary of our methods is provided in Fig 2. We describe the datasets and individual steps taken in detail below.

### Local Expert Disturbance Monitoring Data

Ground based forest monitoring data were provided by local experts employed by the Kafa Zone Bureau of Agriculture. The design of the local disturbance monitoring forms are described in detail in Pratihast et al. (2014) [42]. These forms were designed as reporting tools for local experts to report disturbances (deforestation or forest degradation) or positive forest changes (afforestation or reforestation). We used Open Data Kit (ODK) [46] to integrate these forms with GPS and multimedia (photo, audio). As such, each form contains a range of attributes describing forest status and history and is associated with at least one coordinate pair, five photos (facing north, east, south, west and upwards) and a narrative plot description (input by hand or recorded as audio by the local expert).

**Fig 3:**
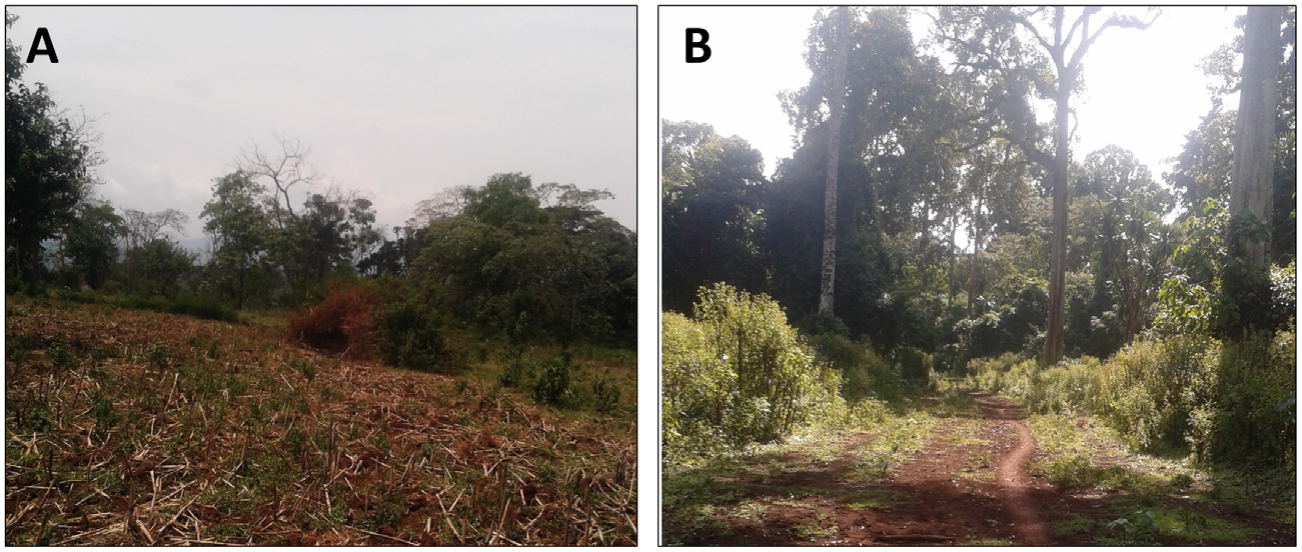
Photo evidence from local disturbance reports documenting deforestation (A) and degradation (B). The location shown in panel A corresponds to the time series shown in Fig 4, and the location shown in panel B corresponds to the time series shown in Fig 5.

We filtered and classified the local disturbance reports into forest change types as shown in S1 Fig. We first assigned a provisional class label (hatched circles in S1 Fig) to the reports automatically, based on the current status of the forest and evidence of previous or ongoing disturbance. For these data to be used in an automated workflow, it was necessary to control for the reliability and consistency of the data [47]. We thus modified the provisional class label where appropriate based on photo evidence (shown in Fig 3), general narrative description of the plot and very high resolution (VHR) imagery from GoogleEarth^™^. After visually validating each form, we assigned the definitive labels of “deforestation”, “degradation”, “no change” or “non-forest”. Given the fact that most reports described deforestation or degradation processes, we finally excluded forms from the other two classes from subsequent analysis. We supplemented the final local expert dataset with randomly sampled and validated no-change pixels from a previous study in the region [13] to ensure both change and no-change classes were sufficiently represented in the dataset used to train the forest change models.

### LTS Pre-processing

We downloaded all available Landsat imagery from the Landsat5-TM, Landsat7-ETM+ and Landsat8-OLI sensors with cloud cover below 80% per scene and processing level L1T from the USGS Earth Explorer system. We selected all available spectral bands except for the thermal band (shown in Table 1). All TM and ETM+ scenes were already processed to surface reflectance level using the Landsat Ecosystem Disturbance Adaptive Processing System (LEDAPS) atmospheric and topographic correction algorithm [48]. OLI scenes were already processed to surface reflectance level by the USGS internal L8SR algorithm. We applied a cloud mask derived from the Function of Mask (FMASK) algorithm [49] to each of the scenes, masking out clouds, cloud shadows and gaps due to the malfunctioning scan-line corrector (SLC) of the ETM+ sensor. Since there were virtually no image acquisitions over our study area during the 1990’s, leaving a large gap in the Landsat archive, we limited our time series to all data after and including 1999, coinciding with the launch of the ETM+ sensor. From a visual screening of all imagery in the archive, we identified cloud pixels frequently missed by the FMASK-derived cloud mask, especially where these clouds coincided with SLC-off gaps in ETM+ images. To reduce the number of these contaminations, we applied a 5-pixel sieve to all images, where pixel clusters surrounded by masked values of five pixels or less were removed from the images. In our assessment, we did not find any significant geo-location errors in the dataset. Since the noise component of the BFAST method [29] can account for occasional outliers due to such errors, we did not carry out any further quality assessment.

**Table 1:** Spectral bands on the Landsat TM, ETM+ and OLI sensors.

Band	Abbreviation	*λ*(TM)	*λ*(ETM+)	*λ*(OLI)
1 (TM/ETM+), 2 (OLI)	B	0.45–0.52μm	0.45–0.52μm	0.45–0.51μm
2 (TM/ETM+), 3 (OLI)	G*	0.52–0.60μm	0.52–0.60μm	0.53–0.59μm
3 (TM/ETM+), 4 (OLI)	R	0.63–0.69μm	0.63–0.69μm	0.64–0.670μm
4 (TM/ETM+), 5 (OLI)	NIR	0.77–0.90μm	0.77–0.90μm	0.85–0.880μm
5 (TM/ETM+), 6 (OLI)	SWIR1	1.55–1.75μm	1.55–1.75μm	1.57–1.65μm
7 (all sensors)	SWIR2*	2.08–2.35μm	2.09–2.35μm	2.11–2.29μm

*Spectral bands used to produce final forest change maps.

Using the pre-processed surface reflectance layers shown in Table 1, we computed a selection of spectral indices, shown in Table 2. These indices have been shown in previous research to be sensitive to vegetation characteristics, states or change dynamics [50–57]. Coefficients for the three basic tasseled cap indices (brightness, greenness and wetness) are shown as *b*, *g* and *w*, respectively, in Table 2. Since data from all sensors were pre-preprocessed to surface reflectance products, we used the same surface reflectance derived tasseled cap coefficients across all sensors [27, 55], which are shown in S1 Table in the Supplemental Materials.

**Table 2:** Spectral indices used in this study.

Name	Abbreviation	Equation	Remarks	Reference(s)
Normalized Difference Vegetation Index	NDVI	$\frac{NIR-R}{NIR+R}$	sensitive to photosynethetic activity	[50]
Normalized Difference Moisture Index	NDMI	$\frac{NIR-SWIR1}{NIR+SWIR1}$	sensitive to canopy moisture content	[51, 52]
Normalized Burn Ratio	NBR	$\frac{NIR-SWIR2}{NIR+SWIR2}$	sensitive to disturbances and fire	[53]
Normalized Burn Ratio 2	NBR2	$\frac{SWIR1-SWIR2}{SWIR1+SWIR2}$	sensitive to disturbances and fire	
Tasseled Cap Brightness	TCB	*b*_1_*B*+*b*_2_*R*+*b*_3_*G*+*b*_4_*NIR*+*b*_5_*SWIR*1 + *b*_6_*SWIR*2	sensitive to surface brightness	[54, 55]
Tasseled Cap Greenness	TCG	*g*_1_*B*+*g*_2_*R*+*g*_3_*G*+*g*_4_*NIR*+*g*_5_*SWIR*1 + *g*_6_*SWIR*2	sensitive to vegetation greenness	[54, 55]
Tasseled Cap Wetness	TCW*	*w*_1_*B* + *w*_2_*R* + *w*_3_*G* + *w*_4_*NIR* + *w*_5_*SWIR*1 + *w*_6_*SWIR*2	sensitive to vegetation moisture content	[52, 54, 55]
Tasseled Cap Angle	TCA	$tan^{-1}\left(\frac{TCB}{TCG}\right)$	sensitive to above-ground biomass	[56, 57]

*Spectral indices used to make final change probability maps.

Since we used all spectral bands and derived indices in our forest change models, we refer to the combination of bands and indices as “spectral bands” for the remainder of this study.

### Deriving Temporal Metrics

Our specific objective in this study was to differentiate between three main forest state classes: deforestation, degradation and no-change. We derived a series of temporal metrics from time series of each of the spectral bands described above and in Tables 1 and 2, recognizing that these forest state classes can involve either gradual or abrupt (i.e. involving a break between adjacent observations) changes. We thus derived temporal metrics which can be divided into two broad categories: (1) full time series and (2) segment-based metrics. We derived these metrics from pixel time series at sites coinciding with local disturbance reports.

For each spectral band, we fit a linear function to the entire time series. We chose the robust linear regression (RLM; [58]) instead of the commonly-used linear regression based on ordinary least squares (OLS). RLM is based on the M-estimator, which seeks to find the best fit to a distribution of data with outliers [58]. This choice of fitting method was motivated by the fact that full Landsat time series commonly contain noise due to unmasked clouds or other sources [13, 19, 59]. The output of this method applied to each time series and spectral band thus consisted of (1) the RLM intercept (using the baseline year 1999 as the origin), and (2) the RLM slope.

While an overall RLM trend can help to describe gradual changes or to discriminate between change and no-change classes, abrupt changes or onset of gradual changes late in a time series may not be sufficiently captured using this method. To describe these changes, we tested each pixel time series for each spectral band for the presence or absence of breaks using the “breakpoints” method of Bai and Perron (2003) [60], which determines the optimal number of breaks in a time series based on the Bayesian Information Criterion (BIC; [60]). We assumed that in the length of the time series (from 1999 to 2015), a land use or land cover change event would occur only once, and were thus interested in identifying the most important break. We therefore set the maximum number of breaks to one, generating a result representing the presence or absence of a break in the time series [61].

**Fig 4:**
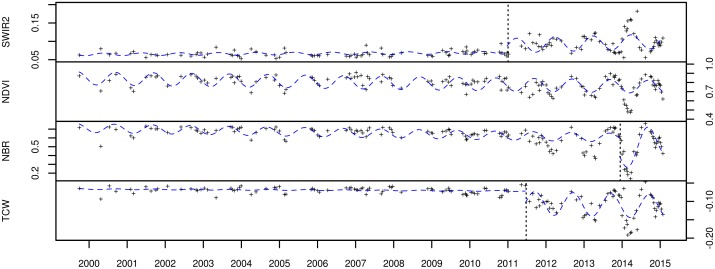
Time series over a deforested site for four spectral bands: SWIR2, NDVI, NBR and TCW. The RLM-fitted season-trend model for each segment is shown as a dotted line. Local disturbance photo evidence for this site is shown in Fig 3A.

**Fig 5:**
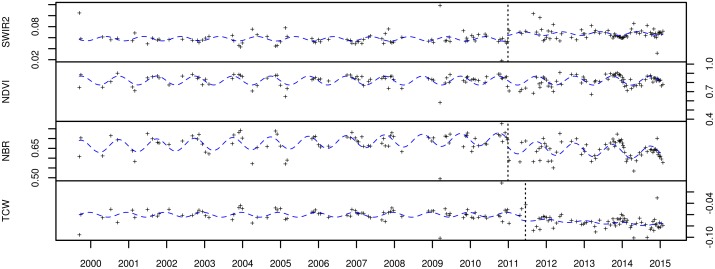
Time series over a degraded forest site for four spectral bands: SWIR2, NDVI, NBR and TCW. The RLM-fitted season-trend model for each segment is shown as a dotted line. Local disturbance photo evidence for this site is shown in Fig 3B.

For each segment that resulted from the breakpoint computation above, we fit season-trend models as in Verbesselt et al. (2010) [29] as follows. For a time-dependent response variable *y*_*t*_, we fit the formula

$$y_{t}=\alpha_{j}+\beta_{j}t+\gamma_{j}sin\left(\frac{2\pi t}{f}+\delta_{j}\right)$$ (1)

where *α*_*j*_ is the intercept, *β*_*j*_ is the linear slope, *γ*_*j*_ is the amplitude, *f* is the frequency of the time series (set to 365 days for LTS data) and *δj* is the phase for each segment *j*. Similarly to the overall trend fitting, we used RLM instead of ordinary least squares (OLS) in fitting the season-trend models. The output of the time series segmented applied to each time series and spectral band thus consisted of (1) the amplitude of the first segment (*γ*_1_), (2) the amplitude of the second segment (*γ*_2_; equal to the first amplitude if no break was detected), (3) the trend of the first segment (*β*_1_) and (4) the trend of the second segment (*β*_2_; equal to the first trend if no break was detected). These outputs are shown for two sites (Fig 3) representing deforestation and degradation in Figs 4 and 5.

### Modelling Forest Change Processes

#### Random Forest Models

We used random forests to model change type as a function of LTS spectral-temporal metrics. The random forest classifier is based on a machine learning algorithm which constructs many decision tree classifiers based on bootstrapped samples [62]. Several advantages of the random forest method over other classifiers have been reported in the literature, including the ability to accommodate many predictor variables, as well as the fact that it is a non-parametric classifier (i.e. does not assume any underlying distribution in the training samples) [62]. Random forest classifications generally assign class labels based on the majority vote among all bootstrapped classification trees. In this study, we used the majority votes from 7000 classification trees to analyze the internal out-of-bag (OOB) error estimates per class. We used the class probabilities (also based on the number of votes per class) to study the impact of the updated local data stream and to map change probabilities at several sites (described below).

#### Iterative Model Updating

Monitoring activities carried out by local experts were carried out in phases according to project activities in the Kafa BR. Specifically, initial trainings were held with local experts to collaboratively develop ODK-based tools for forest monitoring in 2012, after which several rounds of monitoring were carried out until 2014 [42]. From October 2014, a new Integrated Forest Monitoring System (IFMS) was piloted for the Kafa BR with additional trainings in October, a demonstration phase in November and December 2014, and an operational near real-time monitoring phase from January 2015 onwards. While the research described in this paper takes place within the context of this IFMS, the details of the system is the subject of future research in preparation, and is not the focus of this paper.

**Fig 6:**
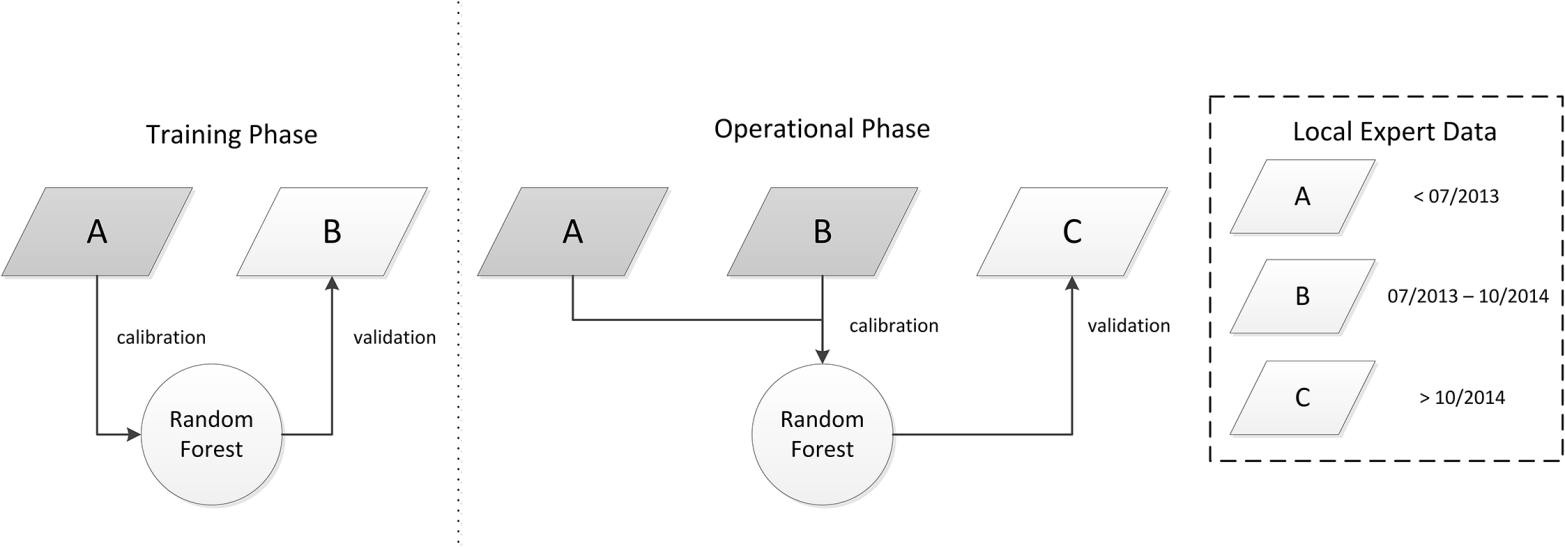
Flowchart demonstrating the iterative updating of random forest models. Time of acquisition of local expert data (parallelograms) are shown in the box on the right hand side. In each phase, a subset of the local expert data were used for model calibration (grey), and another subset was used for model validation (white).

To demonstrate the use of a continuous data-stream from local experts, we ran the random forest algorithm as described above for two time periods: (1) a training phase and (2) an operational phase roughly according to the project phases described above. We divided the local expert data as outlined in Fig 6. During an initial “training” phase, we took all local expert data acquired before July 2013 (“period A” in Fig 6) and used them with all LTS spectral-temporal covariates to train a random forest model as described above. In addition to the OOB error estimate, we used additional local expert data acquired during the period between July 2013 and October 2014 (“period B” in Fig 6) to validate this model. Specifically, we compared the distribution of predicted class probabilities for all disturbance locations reported in period B with the actual change types reported by local experts. During a subsequent “operational” phase, we fused the local expert data from periods A and B, and built a new random forest model using all spectral-temporal covariates. We then used all local expert data acquired after October 2014 (“period C” in Fig 6) to compare predicted class probabilities with actual class labels as in the training phase.

#### Selecting Important Variables

Mapping forest change classes requires that all covariates used in the change type prediction are computed over all pixels in a scene. The computation of breakpoints for each pixel necessary for deriving temporal metrics was computationally time-consuming and not realistic for producing wall-to-wall change maps. We therefore decided to produce maps using simplified random forest models built with only a subset of the most important spectral-temporal covariates. The importance of individual covariates in random forest models is measured either as the mean decrease in accuracy when each variable is removed from the individual bagged decision trees or as a node impurity coefficient [62]. These measures suffer from two possible drawbacks. First, the model composition is different with every run, resulting in different outcomes for every random forest model. Second, when the covariates included in the model are tightly correlated with each other, interpretation of importance can be problematic [63]. For example, if coefficients A and B are both seen to be important predictor variables and are both highly correlated with each other, it is not clear if the importance is due to this correlation or the underlying predictive power of either covariate. To overcome this second drawback, Strobl et al. (2008) [63] proposed a conditional importance measure which involves measuring importance among permuted samples of covariates. With a large number of covariates, however, we found this approach to be computationally unreasonable.

To circumvent this limitation, we derived a scoring algorithm based on iterations of random forests run separately for each spectral band (Tables 1 and 2), using temporal metrics derived from each respective band as model covariates. Specifically, we ran the algorithm 1000 times for all temporal metrics derived from only the blue band, the green band, and so forth for all other spectral bands. For each of the 1000 iterations, we ranked the bands based on the overall accuracy as well as the class-specific accuracies. We then derived a score (*S*) for each band (*j*) and change class (Δ) by taking the average normalized rank over all iterations as follows:

$$S_{j,\Delta }=\frac{1}{N}\sum_{i}^{N}\frac{x_{i,j}-1}{n-1}$$ (2)

where *x*_*i*,*j*_ is the rank of band *j* in iteration *i*, *n* is the total number of spectral bands and *N* is the total number of iterations. *x*_*i*,*j*_ was computed such that the bottom ranking band in iteration *i* was assigned a value of 1, and the top ranking band was assigned a value equal to *n*. Since it is a normalized rank, *S*_Δ_ falls within the interval [0, 1], where a maximum score of one indicates a top rank for all *N* iterations. We selected the most important spectral bands based on this scoring algorithm and produced maps of change class probabilities for several sites. We applied a forest mask produced from a Landsat ETM+ scene acquired on Feburary 2001 to filter out pixels representing stable non-forest from before 2001.

## Results

### Model Accuracies and Temporal Variable Importance

The random forest constructed with 7000 trees using all spectral-temporal covariates and training data gave an overall OOB error estimate of 29%. The deforestation class error was 26%, the degradation class error was 31% and the no-change class error was 32%. Although subsequent iterations of the modeling process showed inconsistencies in importance metrics, the overall RLM trends from various spectral bands were consistently ranked as the most important predictors. The amplitude of the second segment (*γ*_2_) was also frequently highly ranked, followed by trends of the first and second segments (*β*_1_ and *β*_2_).

### Iterative model updating

**Fig 7:**
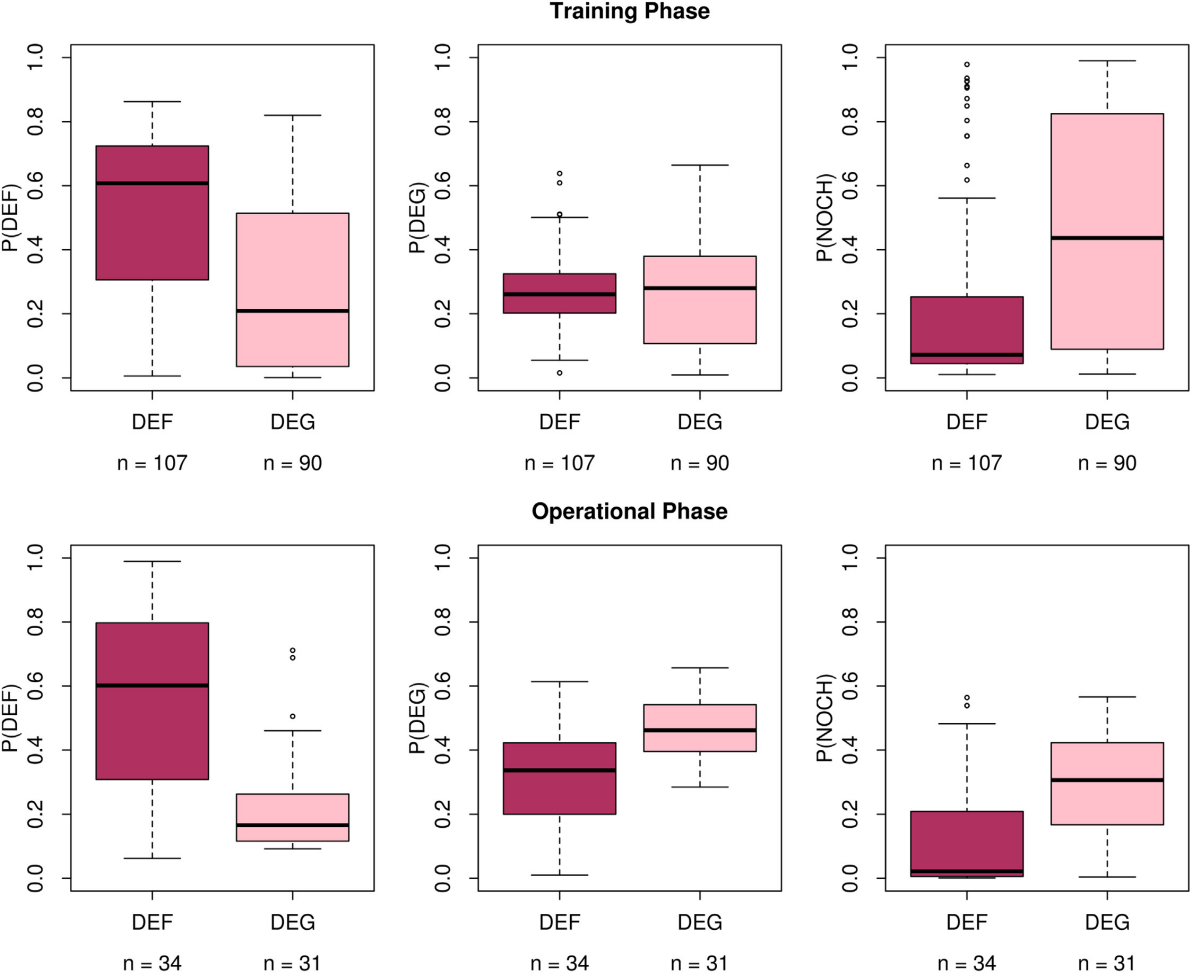
Iterative calibration and validation of change classes. Boxplots of random forest class probabilities for the deforestation (*P*(*DEF*)), degradation (*P*(*DEG*)) or no-change (*P*(*NOCH*)) computed for *in situ* data having DEF or DEG labels are shown for the training phase (top panel) and operational phase (bottom panel) of the monitoring activities. The model updating approach is shown in Fig 6.

The results of the iterative model updating are shown in Fig 7. Here, the class probabilities (P) of deforestation (DEF), degradation (DEG) and no-change (NOCH) are shown for reference deforestation and degradation (DEF and DEG on the x-axis, respectively), both for the training phase (top row) and the operational phase (bottom row). In the training phase, the median class probabilities for reference deforestation locations were 61% for deforestation, 26% for degradation and 7% for no change. These class probabilities remained largely static during the operational phase: 60% for deforestation, 34% for degradation and 2% for no change.

The median class probabilities for reference degradation locations in the training phase were 21% for deforestation, 28% for degradation and 44% for no change. These probabilities changed to 17% for deforestation, 46% for degradation and 31% for no change during the operational period.

### Importance of spectral bands in classifying change types

**Fig 8:**
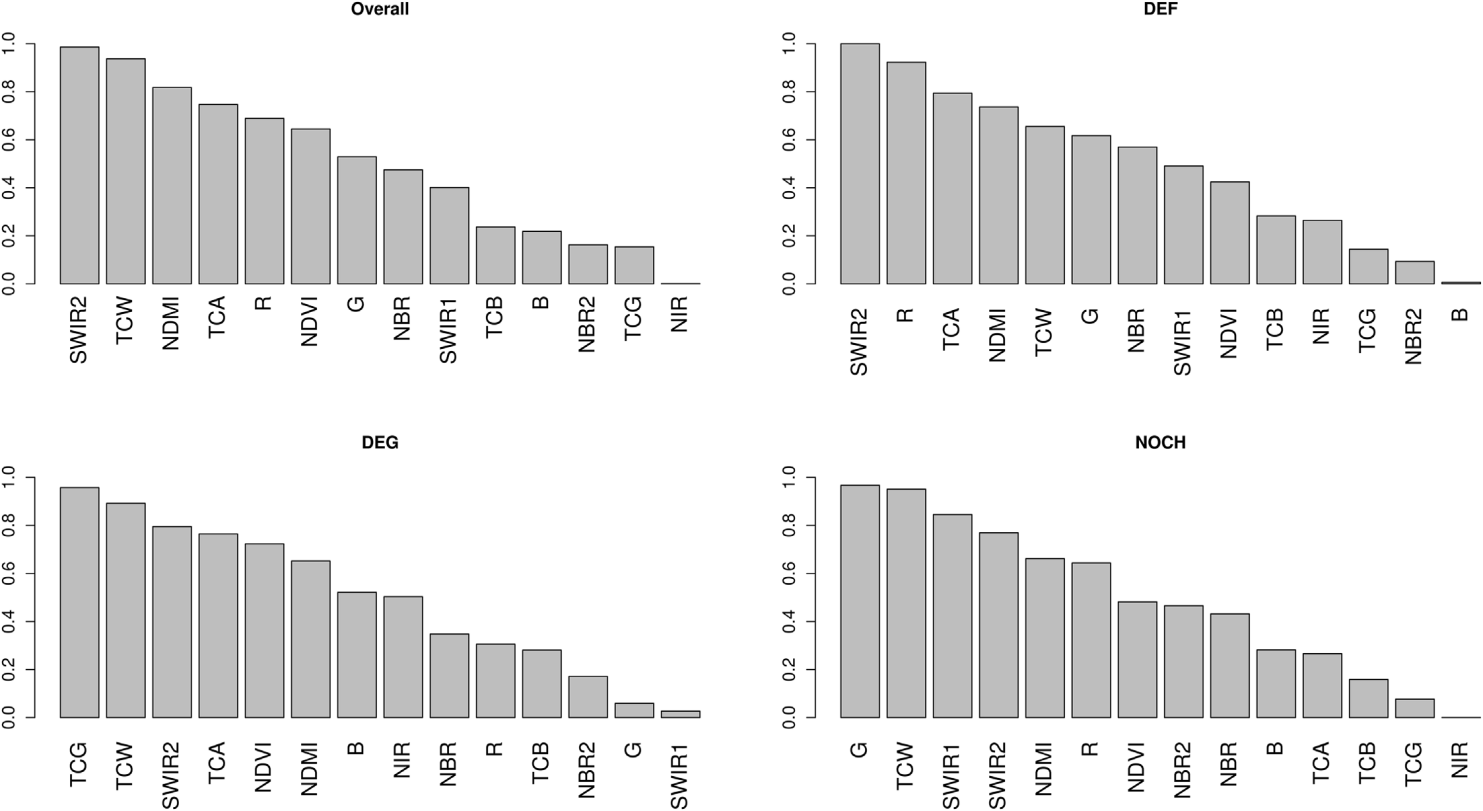
Importance scores (*S*) for each band based on overall accuracies and class accuracies for deforestation (DEF), degradation (DEG) and no-change (NOCH).

The importance scores for each spectral band are shown in Fig 8. SWIR2 and TCW emerged as the most important variables when overall accuracies were considered (i.e. taking all change classes into account). The most important bands for the individual change classes were SWIR2 for deforestation, TCG for degradation and G for no-change. SWIR2 achieved a perfect score of 1 for the deforestation class, implying that it was ranked the highest in terms of deforestation accuracy on every iteration. The NIR band, on the other hand, received a score of zero for the no-change class, implying that it was consistently the lowest ranked band for that class over all iterations.

### Spatial distribution of deforestation and degradation

**Fig 9:**
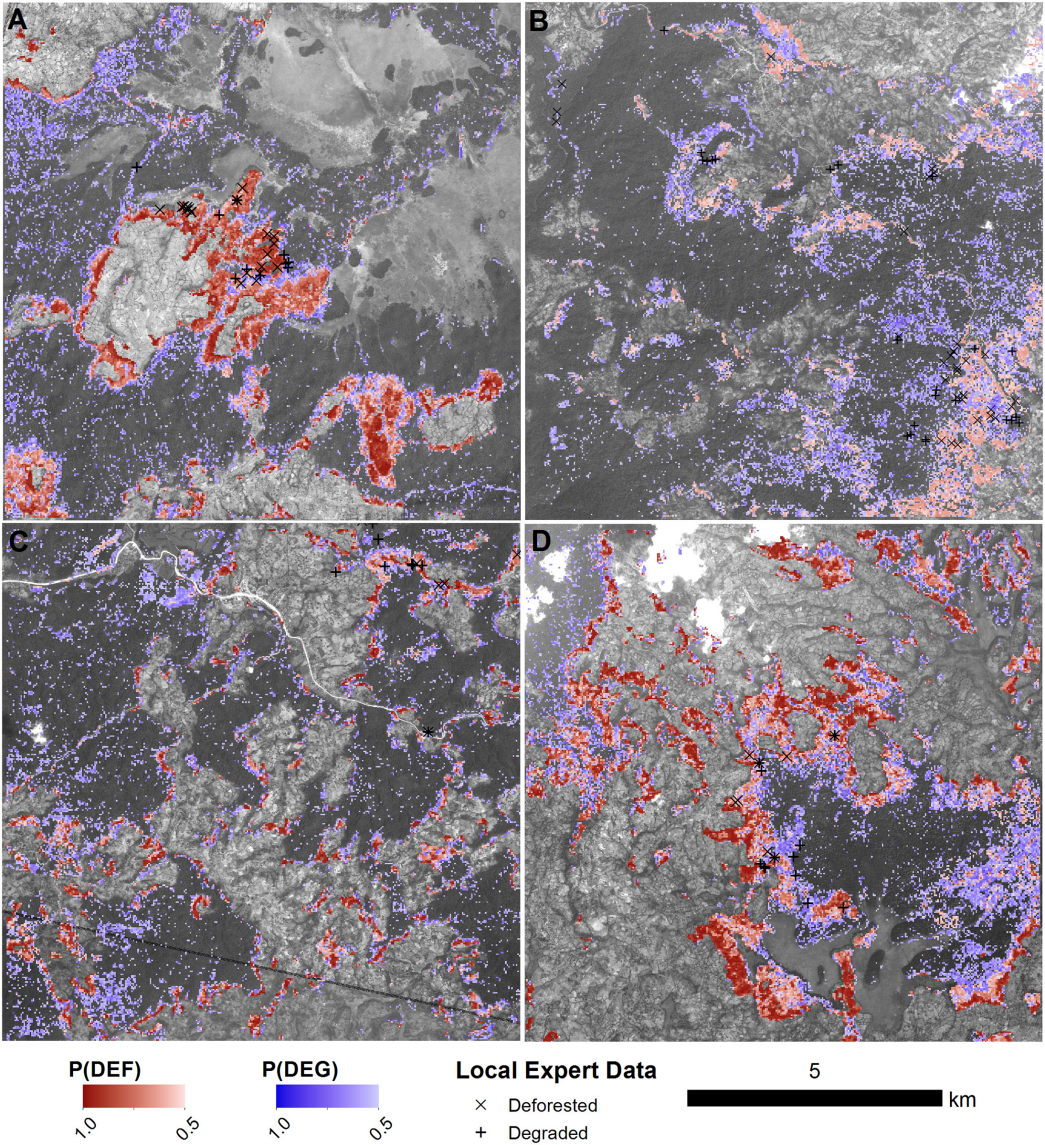
Maps of deforestation and degradation at four sites. The probability of deforestation and degradation are shown as red and blue colour maps, respectively. Local expert reports of deforestation (X) or degradation (+) collected between 2012 and 2015 are overlaid on the maps. The base images are SPOT5 images (band 2; 2.5m spatial resolution) acquired between 2009 and 2011. Dark shaded areas represent forest in the SPOT5 image, and light areas are non-forest land cover types (e.g. cropland or wetland). The locations of each tile (A to D) are shown in Fig 1.

**Fig 10:**
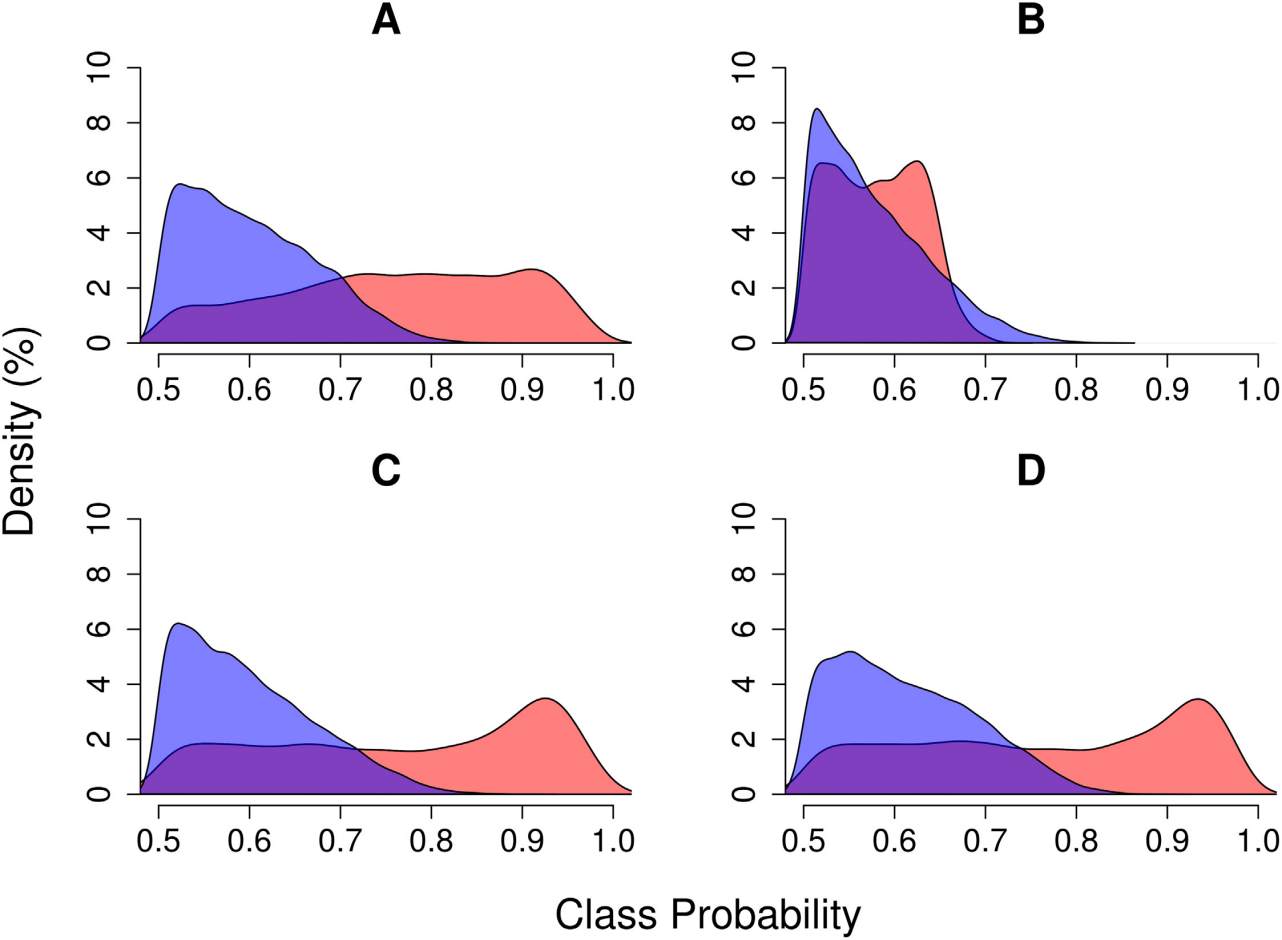
Random forest class probability histograms for deforestation (red) and degradation (blue) at each of the four sites shown in Fig 9.

Based on the results of the importance scoring algorithm, we selected all temporal covariates derived from the SWIR2 and TCW bands for further analysis. We additionally selected the RLM intercept and trend of the green band (G) based on its apparent importance in discriminating stable forest (no-change). Using this subset of the covariates, we derived another random forest model. The overall class error of the revised random forest model was 28%, with class errors of 23% and 33% for deforestation and degradation, respectively. We produced maps of change type probabilities (deforestation, degradation and no-change). Maps of forest change probabilities (deforestation or degradation) for four sites are shown in Fig 9, and histograms of deforestation and degradation class probabilities for each site are shown in Fig 10. In general, deforestation was mapped with high certainty. Degradation, on the other hand, was spatially diffuse and class probabilities were generally lower than that of deforestation. One of the four sites (Figs 9B and 10B) had noticeably lower deforestation probabilities, despite the fact that an abundance of local expert data confirmed the deforestation events at that site.

## Discussion

### Detecting changes using an integrated approach

Our first research question concerned the ability to distinguish deforestation and degradation in our study area using local expert data in combination with LTS. In the current study, we provide evidence that deforestation and degradation are indeed separable using a random forest approach, with OOB class accuracies for both deforestation and degradation on order of disturbance accuracies reported previously [13]. Two main factors contributed to this ability. While a direct comparison between methods and results is difficult, a similar study conducted in the same area achieved similar accuracies in disturbance monitoring using LTS (73% user’s and producer’s accuracies) [13]. Similarly to DeVries et al. (2015) [13], we were able to track small-scale deforestation (Fig 9), with the key difference that our models were able to predict degradation above a 50% probability threshold in many cases. Using NDVI time series and an Ordinary Logistic Regression approach, DeVries et al. (2015) [13] were unable to achieve predicted class probabilities above 25% for degradation, precluding the mapping of degraded forest with certainty. In Fig 9, on the other hand, we demonstrate how degradation can be mapped alongside deforestation when prediction probabilities are sufficiently high.

Improvements in sensitivity to forest degradation are owed in part to our overall workflow. Most other remote sensing method-related studies validate an existing method using ground-data or visually interpreted data sampled from an existing map or result, and indeed use local expert or community-based monitoring (CBM) data to validate samples selected from existing results [64]. In this study, however, we used *a priori* training data to help to develop the method itself. These existing data underscore the value of the local expert data stream featured in this study. This bottom-up approach was especially important for examining the extent to which we could track forest degradation, since local experts were able to identify cases of below-canopy disturbances independently of any remote sensing based datasets. Notably, with this approach we found that SWIR-based indices are consistently more sensitive to changes in our study area than indices based on the NIR or visible wavelengths, such as NDVI [13].

The flexibility of our bottom-up integration approach can be extended to land cover changes not explored in this study. We assumed that significant land cover changes occur a maximum of one time during a 16-year time series. While this assumption is generally reasonable for Southern Ethiopia, where small-holder agriculture drives deforestation and tends towards permanent agriculture [65], it does not hold true for other shifting agricultural systems, where multiple disturbance-recovery cycles would be expected in the time series [66]. Our approach can still be tuned to such cases, whereby *in situ* forest state observations (“deforested” or “degraded” in this study) can be used to classify such patterns based on their spectral-temporal signatures.

### A continuous local data stream

The local expert data used in this study were generated as a result of a series of field trainings, local monitoring activities [42] and the development of an Integrated Forest Monitoring System (IFMS) involving local experts (forest rangers) in the Kafa BR. We divided the dataset into a training and operational phase to represent this process and then compared reference data from each period with predicted class probabilities derived from iteratively trained random forest models. While the median class probabilities between the two phases did not differ substantially, the spread of probabilities showed a marked change from a wide spread in the training phase (Fig 7, top row) to more narrow distributions in the operational phase (Fig 7, bottom row). Most importantly, the apparent confusion between degradation and no-change classes in the training phase was reduced as evidenced by the generally lower no-change probabilities among degradation reference samples in the operational phase, an important prerequisite to mapping degradation with a degree of certainty (Fig 9).

Two possible factors may influence the improvements in estimated class probabilities seen in Fig 7. First, the absolute number of training samples available with subsequent monitoring phases likely have a favourable effect on the random forest models. DeVries et al. (2015) [13] found that degradation samples were associated with and without time series breakpoints and a range of change magnitude values. For this reason, an increase in the number of training samples provides a better range of degradation “states” from which to train the models, particularly considering the fact that local experts are more able to identify degradation from the ground than is possible with optical remote sensing data [42]. Second, it is possible that the quality of the local expert data increase over time as they become more experienced with the monitoring tools and receive subsequent follow-up trainings [38, 42]. Notably, the last phase corresponds with the kick-off of an IFMS, which is intended to provide a platform for local stakeholders to share local data, experiences and gain access to satellite-based forest change alerts. Such a system is expected to spur increased and enhanced local monitoring data, allowing for further development and testing of forest change models.

### Mapping deforestation and deforestation

The Kafa BR, like many other areas in the tropics, is characterized by forest mosaic landscapes and complex deforestation and degradation patterns [13, 16]. Characterizing and mapping these processes is therefore important to understanding forest changes. The spatial distribution of deforestation and degradation shows that deforestation and degradation exhibit logical spatial patterns. Previous research in the Kafa BR has shown that deforestation occurs at small scales [13]. In general, we found that deforestation probabilities were generally quite high in these change areas, confirming that LTS is a suitable data source for tracking small-scale deforestation [13]. Given the fact that the drivers of degradation in the Kafa BR are tightly associated with deforestation [42], it is not surprising that the areas with relatively high degradation probabilities seem to be associated with deforestation fronts. This mapping approach can thus be used for at least two key purposes. First, deforestation and especially degradation hotspots can be used to alert local stakeholders and monitoring experts of areas with possible disturbances via an interactive monitoring system. Second, these hotspots can be used for activity-based stratification of the area for measuring biomass, biodiversity or other important ecological variables.

Despite the quality of the maps produced, it should be noted that deforestation class probabilities were markedly lower at one of our sites than in the other test sites (Fig 9B). This particular site is located near the edge of the Landsat scene used in this study, a region where data availability is known to be limiting [13]. It is possible that higher uncertainties in the deforestation class are a result of a relative lack of observations in the LTS dataset, which can preclude the fitting of a reliable seasonal model. With a sub-standard season model, the seasonal amplitude of either segment cannot be reliably estimated, causing errors in the deforestation class. Considerations must therefore be made for data availability when choosing temporal variables with which to model forest change.

### Limitations to the method

A number of limitations to the approach used in this study are discussed in this section, including definitions of forest degradation, sampling considerations and the timing of forest changes.

#### Definition of forest degradation

Making a distinction between deforestation and forest degradation processes is problematic when dealing with the complex forest change processes encountered in this study. While we systematically distinguished between these two processes among local expert disturbance reports, comparison with LTS profiles reveals a more subtle distinction between these processes. Characterizing a disturbance “event” is complicated by the fact that deforestation is preceded by several years of forest degradation when driven by subsistence agriculture [13, 42]. Our classification scheme could thus be alternatively viewed as “state variables”, in which forest pixels at a given point in time were classified as “deforested” or “degraded” depending on a suite of spectral-temporal variables. Given difficulties with defining degradation [45], a more practical definition of degradation would be a continuous measurable value [43]. To this end, our approach could be expanded to include other tools, such as hemispherical photography [67, 68] to provide a continuous measure of canopy cover over monitored sites. Additionally, expanding the local monitoring activities to include regular biomass measurement campaigns [35, 38] would provide additional continuous forest variables to describe forest change. Even with a quantitative measure of forest change, however, any approach using LTS data to map degradation is practically bound by changes to the forest canopy. Other forms of forest degradation, including alteration of ecosystem function by lianas [69–71] or other invasive species [72], are unlikely to be captured by LTS spectral-temporal covariates if the canopy itself is not sufficiently impacted. In such cases, the *in situ* data stream discussed here plays an important role as an indicator of where such types of degradation are occurring and not being sufficiently detected by LTS data.

#### Sampling considerations

The data used in the the iterative validation of the random forest models were not probabilistically sampled, but were rather based on purposive observations by local experts. As can be expected from CBM-based data streams, local expert data were limited to locations that were accessible to the forest rangers in this study. Accessibility limitations are not only limited to the spatial distribution of local expert observations, but also to the timing and frequency of observations [42]. Prescribing plot locations, on the other hand, could lead to the temptation to either approximate site locations (negatively affecting the spatial data quality) or to forgo monitoring altogether (negatively affecting the data stream). A purposive sampling design was therefore necessary for preserving the quality and quantity of the local expert data collected in this study.

Despite drawbacks in sampling and validation, the internal OOB sample provided by the random forest models provide an alternative robust measure of model accuracy. Additionally, we demonstrated the ability of continuously acquired local expert data to improve and validate the random forest models over time (Fig 7). The maps produced by these models (Fig 9) can be used to support the continuous training and validation of random forest models by directing local experts to locations with high probability of forest change.

#### Timing of changes

The timing of change is an important feature of forest monitoring, for which reference data often consist of visually interpreted imagery [41]. Even though local experts also record disturbance timing, we did not attempt to model change timing in this study due especially to uncertainties in the local expert data. These uncertainties arose largely because of the way in which change types and onset times are defined. Pratihast et al. (2014) [42] found that temporal discrepancies between change times recorded by local experts and those observed using very high resolution imagery arose because of two possible differences. First, local rangers are able to detect understorey degradation before this is visible to satellite sensors, causing a temporal lag on the side of the satellite data. Second, local experts tended to define deforestation in terms of land use, implying that preceding degradation activities (e.g. for fuelwood and timber harvesting or understorey coffee cultivation) were not interpreted as deforestation, causing a temporal lag on the side of the local experts [42]. To avoid confusion in our change models, we decided therefore to focus on the thematic dimension of forest change.

#### Continuity and Consistency of Time Series

The continuity of the Landsat observation record is the motivation behind the launch of the eighth Landsat sensor in 2013 [73]. Since some part of the Kafa BR are known to be Landsat data poor [13], the addition of OLI data at the end of the LTS is seen as a distinct advantage in this study. The spectral resolution (Table 1) and radiometric resolution (higher bit depth than that of Landsat 7 and 5) are two major differences between Landsat 8 and its predecessors that were not taken into account in this study, however. Research has shown that despite a difference in dynamic range of NIR spectral reflectance values between OLI and ETM+ data, surface reflectance and derived metrics do not differ significantly between sensors [74]. Further research into the cross-sensor comparability and need for normalization for other systems and objectives is still needed, however. Specifically, significant differences in spectral reflectance could have an impact on class predictions made in this study.

#### Overcoming limitations using Integrated Forest Monitoring

Limitations in sampling and change timing could be addressed by further exploring the idea of an interactive monitoring system between local experts and remote sensing specialists. Future research is aimed at demonstrating an operational IFMS for the Kafa BR that is designed to support such ongoing monitoring activities. Such research should further investigate how the forest change outputs of our method can be used in an interactive environment to support follow-up monitoring, management and enforcement, including the temporal dimension of change in the context of a near real-time interactive monitoring system, for example.

## Conclusions

In this study, we have provided the first demonstration of local expert forest monitoring data integrated with Landsat Time Series (LTS) using a machine learning (random forest) approach. We found that local expert monitoring data and dense LTS are valuable in training and validation random forest models to predict deforestation and degradation in complex forest matrix landscapes. Notably, we showed that as local expert monitoring data continued to be collected and received, model results improved, demonstrating the potential of an ongoing forest monitoring system featuring both data streams. From the models, we determined that the SWIR2 and TCW spectral bands were the most important for differentiating deforestation and degradation, and used temporal covariates based on these bands to produce spatial predictions of forest change. This study provides a basis on which further research on integrated forest monitoring systems, particularly those seeking to integrate community-based monitoring (CBM) or volunteered geo-information (VGI) data with dense satellite time series. Future research will follow-up on our approach by incorporating other data sources using data fusion methods [75], such as Sentinel-2, terrestrial or airborne LiDAR or other airborne remote sensing datasets. Furthermore, our approach is flexible to the types of predictions and can include other types of forest change as needed, such as afforestation and reforestation activities.

## Supporting Information

S1 Fig Local expert data pre-precessing workflow. Decision flowchart for classification of local disturbance reports. The primary class labels deforestation (DEF), degradation (DEG), no change (stable forest) or non-forest were assigned based on automatic interpretation of form attributes (circles with hatched outlines). Primary class labels were then verified using plot photos, plot descriptions and very high resolution (VHR) satellite imagery and final class labels (circles with solid outlines) were assigned.
(TIFF)

S1 Table Tasseled Cap coefficients for surface reflectance data. Coefficients used to transform reflectance bands from all Landsat sensors into tasseled cap indices.
(PDF)

S2 Table Random forest importance scores. Random forest scores based on overall accuracies and class accuracies for deforestation (DEF), degradation (DEG), no-change (NOCH). Spectral bands are shown from highest to lowest overall importance scores.
(PDF)

## References

[1] Gullison RE, Frumhoff PC, Canadell JG, Field CB, Nepstad DC, Hayhoe K, et al Tropical forests and climate policy. Science. 2007;316(5827):985–986. Available from: http://www.globalcarbonproject.org/global/pdf/pep/Post2006/Gullison.2007.DeforestationAll.Science.pdf. 10.1126/science.1136163 17495140

[2] van der Werf GR, Morton DC, DeFries RS, Olivier JGJ, Kasibhatla PS, Jackson RB, et al CO2 emissions from forest loss. Nature Geoscience. 2009;2(11):737–738. 10.1038/ngeo671

[3] Laurance WF, Useche DC, Rendeiro J, Kalka M, Bradshaw CJa, Sloan SP, et al Averting biodiversity collapse in tropical forest protected areas. Nature. 2012 9;489(7415):290–4. Available from: http://www.ncbi.nlm.nih.gov/pubmed/22832582. 10.1038/nature11318 22832582

[4] DeFries R, Hansen A, Newton AC, Hansen MC. Increasing Isolation of Protected Areas in Tropical Forests Over the Past Twenty Years. Ecological Applications. 2005 2;15(1):19–26. Available from: http://www.esajournals.org/doi/abs/10.1890/03-5258. 10.1890/03-5258

[5] Aerts R, Berecha G, Honnay O. Protecting coffee from intensification. Science. 2015;347(6218):139 10.1126/science.347.6218.139-b 25574013

[6] Angelsen A. Realising REDD+: National strategy and policy options. Brockhaus M, Kanninen M, Sills E, Sunderlin WD, Wertz-Kanounnikoff S, editors. Cifor; 2009.

[7] Herold M, Skutsch M. Monitoring, reporting and verification for national REDD + programmes: two proposals. Environmental Research Letters. 2011 1;6(1):014002 Available from: http://stacks.iop.org/1748-9326/6/i=1/a=014002?key=crossref.b8a06ec5da341082aacb4442178ff46f. 10.1088/1748-9326/6/1/014002

[8] DeVries B, Herold M. The Science of Measuring, Reporting and Verification (MRV) In: Lyster R, MacKenzie C, McDermott C, editors. Law, Tropical Forests and Carbon: The Case of REDD+. Cambridge: Cambridge Univ Press; 2013 p. 151–183.

[9] Penman J, Gytarsky M, Hiraishi T, Krug T, Kruger D, Pipatti R, et al Good Practice Guidance for Land Use, Land-Use Change and Forestry. Intergovernmental Panel on Climate Change (IPCC); 2003.

[10] De Sy V, Herold M, Achard F, Asner GP, Held A, Kellndorfer J, et al Synergies of multiple remote sensing data sources for REDD+ monitoring. Current Opinion in Environmental Sustainability. 2012 10;p. 1–11. Available from: http://linkinghub.elsevier.com/retrieve/pii/S1877343512001200.

[11] Coppin P, Jonckheere I, Nackaerts K, Muys B, Lambin E. Digital change detection methods in ecosystem monitoring: a review. International Journal of Remote Sensing. 2004 5;25(9):1565–1596. Available from: http://www.informaworld.com/openurl?genre=article&doi=10.1080/0143116031000101675&magic=crossref||D404A21C5BB053405B1A640AFFD44AE3. 10.1080/0143116031000101675

[12] Romijn E, Herold M, Kooistra L, Murdiyarso D, Verchot L. Assessing capacities of non-Annex I countries for national forest monitoring in the context of REDD+. Environmental Science & Policy. 2012 5;19–20:33–48. Available from: http://linkinghub.elsevier.com/retrieve/pii/S1462901112000202. 10.1016/j.envsci.2012.01.005

[13] DeVries B, Verbesselt J, Kooistra L, Herold M. Robust monitoring of small-scale forest disturbances in a tropical montane forest using Landsat time series. Remote Sensing of Environment. 2015;161:107–121. Available from: http://linkinghub.elsevier.com/retrieve/pii/S0034425715000656. 10.1016/j.rse.2015.02.012

[14] Tyukavina a, Stehman SV, Potapov PV, Turubanova Sa, Baccini a, Goetz SJ, et al National-scale estimation of gross forest aboveground carbon loss: a case study of the Democratic Republic of the Congo. Environmental Research Letters. 2013 12;8(4):044039 Available from: http://stacks.iop.org/1748-9326/8/i=4/a=044039?key=crossref.4eca15f39b9f622768a2962d1b59ca5f. 10.1088/1748-9326/8/4/044039

[15] Thompson ID, Guariguata MR, Okabe K, Bahamondez C, Nasi R, Heymell V, et al An operational framework for defining and monitoring forest degradation. Ecology and Society. 2013;18(2). Available from: http://search.ebscohost.com/login.aspx?direct=true&profile=ehost&scope=site&authtype=crawler&jrnl=17083087&AN=91274450&h=jFp4WPKnOXFKZWxJCmGdPSlU69Qjk76YD8+YBFS+SZbMzEJZVuoh//Mf4wnefR+91ECwGQmEyWMBBZXx8SnVeg==&crl=c. 10.5751/ES-05443-180220

[16] Mertz O, Müller D, Sikor T, Hett C, Heinimann A, Castella C, et al The forgotten D: challenges of addressing forest degradation in complex mosaic landscapes under REDD. 2012;(10):37–41.

[17] Banskota A, Kayastha N, Falkowski MJ, Wulder MA, Froese RE, White JC, et al Forest monitoring using Landsat time-series data- A review. Canadian Journal of Remote Sensing. 2014;40(5):1–37. 10.1080/07038992.2014.987376

[18] Wulder Ma, Masek JG, Cohen WB, Loveland TR, Woodcock CE. Opening the archive: How free data has enabled the science and monitoring promise of Landsat. Remote Sensing of Environment. 2012 7;122:2–10. Available from: http://linkinghub.elsevier.com/retrieve/pii/S003442571200034X. 10.1016/j.rse.2012.01.010

[19] Zhu Z, Woodcock CE, Olofsson P. Continuous monitoring of forest disturbance using all available Landsat imagery. Remote Sensing of Environment. 2012 7;122:75–91. Available from: http://linkinghub.elsevier.com/retrieve/pii/S0034425712000387. 10.1016/j.rse.2011.10.030

[20] Reiche J, de Bruin S, Hoekman D, Verbesselt J, Herold M. A Bayesian Approach to Combine Landsat and ALOS PALSAR Time Series for Near Real-Time Deforestation Detection. Remote Sensing. 2015;7(5):4973–4996. Available from: http://www.mdpi.com/2072-4292/7/5/4973/. 10.3390/rs70504973

[21] Dutrieux LP, Verbesselt J, Kooistra L, Herold M. Monitoring forest cover loss using multiple data streams, a case study of a tropical dry forest in Bolivia. ISPRS Journal of Photogrammetry and Remote Sensing. 2015;Available from: http://linkinghub.elsevier.com/retrieve/pii/S0924271615000970. 10.1016/j.isprsjprs.2015.03.015

[22] INPE. Projeto PRODES: Monitoramento da Floresta Amazônica Brasileira por Satélite; 2014. Available from: obt.inpe.br/prodes/index.php.

[23] INPE. Detecção de Desmatamento em Tempo Real; 2014. Available from: obt.inpe.br/deter/.

[24] Amazon Conservation Association. Monitoring of the Andean Amazon Project (MAAP);. Available from: http://www.maaproject.org/.

[25] World Resources Institute. Global Forest Watch; 2014. Available from: http://www.globalforestwatch.org/.

[26] Hansen MC, Potapov PV, Moore R, Hancher M, Turubanova Sa, Tyukavina a, et al High-resolution global maps of 21st-century forest cover change. Science (New York, NY). 2013 11;342(6160):850–3. Available from: http://www.ncbi.nlm.nih.gov/pubmed/24233722. 10.1126/science.1244693 24233722

[27] Kennedy RE, Yang Z, Cohen WB. Detecting trends in forest disturbance and recovery using yearly Landsat time series: 1. LandTrendr—Temporal segmentation algorithms. Remote Sensing of Environment. 2010 12;114(12):2897–2910. Available from: http://linkinghub.elsevier.com/retrieve/pii/S0034425710002245. 10.1016/j.rse.2010.07.008

[28] Huang C, Goward SN, Masek JG, Thomas N, Zhu Z, Vogelmann JE. An automated approach for reconstructing recent forest disturbance history using dense Landsat time series stacks. Remote Sensing of Environment. 2010 1;114(1):183–198. Available from: http://linkinghub.elsevier.com/retrieve/pii/S0034425709002685. 10.1016/j.rse.2009.08.017

[29] Verbesselt J, Hyndman R, Zeileis A, Culvenor D. Phenological change detection while accounting for abrupt and gradual trends in satellite image time series. Remote Sensing of Environment. 2010 12;114(12):2970–2980. Available from: http://linkinghub.elsevier.com/retrieve/pii/S0034425710002336. 10.1016/j.rse.2010.08.003

[30] Jamali S, Jönsson P, Eklundh L, Ardö J, Seaquist J. Remote Sensing of Environment Detecting changes in vegetation trends using time series segmentation. Remote Sensing of Environment. 2015;156:182–195.

[31] Zhu Z, Woodcock CE. Continuous change detection and classification of land cover using all available Landsat data. Remote Sensing of Environment. 2014 3;144:152–171. Available from: http://linkinghub.elsevier.com/retrieve/pii/S0034425714000248. 10.1016/j.rse.2014.01.011

[32] Conrad CC, Hilchey KG. A review of citizen science and community-based environmental monitoring: Issues and opportunities. Environmental Monitoring and Assessment. 2011;176(1–4):273–291. 10.1007/s10661-010-1582-5 20640506

[33] Boissière M, Beaudoin G, Hofstee C, Rafanoharana S. Participating in REDD+ Measurement, Reporting, and Verification (PMRV): Opportunities for local people? Forests. 2014;5(8):1855–1878. 10.3390/f5081855

[34] Skutsch M, Turnhout E, Vijge MJ, Herold M, Wits T, Den Besten JW, et al Options for a national framework for benefit distribution and their relation to community-based and national REDD+ monitoring. Forests. 2014;5(7):1596–1617. 10.3390/f5071596

[35] Pratihast A, Herold M, Avitabile V, de Bruin S, Bartholomeus H, C Jr, et al Mobile Devices for Community-Based REDD+ Monitoring: A Case Study for Central Vietnam. Sensors. 2012 12;13(1):21–38. Available from: http://www.mdpi.com/1424-8220/13/1/21/. 10.3390/s130100021 23344371 PMC3574662

[36] Foody GM, Boyd DS. Using volunteered data in land cover map validation: Mapping west African forests. IEEE Journal of Selected Topics in Applied Earth Observations and Remote Sensing. 2013;6(3):1305–1312. 10.1109/JSTARS.2013.2250257

[37] Delbart N, Beaubien E, Kergoat L, Le Toan T. Comparing land surface phenology with leafing and flowering observations from the PlantWatch citizen network. Remote Sensing of Environment. 2015;160:273–280. Available from: http://linkinghub.elsevier.com/retrieve/pii/S0034425715000218. 10.1016/j.rse.2015.01.012

[38] Brofeldt Sr, Theilade I, Burgess ND, Danielsen F, Poulsen MK, Adrian T, et al Community monitoring of carbon stocks for REDD+: Does accuracy and cost change over time? Forests. 2014;5(8):1834–1854. 10.3390/f5081834

[39] Ferster CJ, Coops NC. Integrating volunteered smartphone data with multispectral remote sensing to estimate forest fuels. International Journal of Digital Earth. 2015;(2):1–26. Available from: http://www.tandfonline.com/doi/abs/10.1080/17538947.2014.1002865.

[40] Bigagli L, Santoro M, Mazzetti P, Nativi S. Architecture of a Process Broker for Interoperable Geospatial Modeling on the Web. ISPRS International Journal of Geo-Information. 2015;4(2):647–660. Available from: http://www.mdpi.com/2220-9964/4/2/647/. 10.3390/ijgi4020647

[41] Cohen WB, Yang Z, Kennedy R. Detecting trends in forest disturbance and recovery using yearly Landsat time series: 2. TimeSync—Tools for calibration and validation. Remote Sensing of Environment. 2010 12;114(12):2911–2924. Available from: http://linkinghub.elsevier.com/retrieve/pii/S0034425710002269. 10.1016/j.rse.2010.07.010

[42] Pratihast A, DeVries B, Avitabile V, de Bruin S, Kooistra L, Tekle M, et al Combining Satellite Data and Community-Based Observations for Forest Monitoring. Forests. 2014 10;5(10):2464–2489. Available from: http://www.mdpi.com/1999-4907/5/10/2464/. 10.3390/f5102464

[43] Lambin EF. Monitoring forest degradation in tropical regions by remote sensing: some methodological issues. Global Ecology and Biogeography. 1999 5;8(3–4):191–198. Available from: http://doi.wiley.com/10.1046/j.1365-2699.1999.00123.x. 10.1046/j.1365-2699.1999.00123.x

[44] Hirschmugl M, Steinegger M, Gallaun H, Schardt M. Mapping Forest Degradation due to Selective Logging by Means of Time Series Analysis: Case Studies in Central Africa. Remote Sensing. 2014 1;6(1):756–775. Available from: http://www.mdpi.com/2072-4292/6/1/756/. 10.3390/rs6010756

[45] Morales-Barquero L, Skutsch M, Jardel-Peláez E, Ghilardi A, Kleinn C, Healey J. Operationalizing the Definition of Forest Degradation for REDD+, with Application to Mexico. Forests. 2014 7;5(7):1653–1681. Available from: http://www.mdpi.com/1999-4907/5/7/1653/. 10.3390/f5071653

[46] Anokwa Y, Hartung C, Brunette W. Open Source Data Collection in the Developing World. Computer. 2009;42(10):97–99. 10.1109/MC.2009.328

[47] Comber A, See L, Fritz S, Van der Velde M, Perger C, Foody G. Using control data to determine the reliability of volunteered geographic information about land cover. International Journal of Applied Earth Observation and Geoinformation. 2013 8;23:37–48. Available from: http://linkinghub.elsevier.com/retrieve/pii/S0303243412002358. 10.1016/j.jag.2012.11.002

[48] Vermote EF, Deuzé JL, Herman M, Morcrette JJ. Second simulation of the satellite signal in the solar spectrum, 6S: An overview. IEEE Transactions on Geoscience and Remote Sensing. 1997;35(3):675–686. Available from: http://ieeexplore.ieee.org/xpls/abs_all.jsp?arnumber=581987. 10.1109/36.581987

[49] Zhu Z, Woodcock CE. Object-based cloud and cloud shadow detection in Landsat imagery. Remote Sensing of Environment. 2012 3;118:83–94. Available from: http://linkinghub.elsevier.com/retrieve/pii/S0034425711003853. 10.1016/j.rse.2011.10.028

[50] Tucker CJ. Red and photographic infrared linear combinations for monitoring vegetation. 1979;150:127–150.

[51] Wilson EH, Sader Sa. Detection of forest harvest type using multiple dates of Landsat TM imagery. Remote Sensing of Environment. 2002 6;80(3):385–396. Available from: http://linkinghub.elsevier.com/retrieve/pii/S0034425701003182. 10.1016/S0034-4257(01)00318-2

[52] Jin S, Sader Sa. Comparison of time series tasseled cap wetness and the normalized difference moisture index in detecting forest disturbances. Remote Sensing of Environment. 2005 2;94(3):364–372. Available from: http://linkinghub.elsevier.com/retrieve/pii/S0034425704003414. 10.1016/j.rse.2004.10.012

[53] Key CH, Benson NC. Landscape assessment: Ground measure of severity, the composite burn index; and remote sensing of severity, the Normalized Burn Ratio FIREMON: Fire effects monitoring and inventory system, USDA Forest Service General Technical Report RMRS-GTR-164-CD. Fort Collins, CO: USDA Forest Service Rocky Mountain Research Station; 2006.

[54] Crist EP, Cicone RC. A Physically-Based Transformation of Thematic Mapper Data—The TM Tasseled Cap. IEEE Transactions on Geoscience and Remote Sensing. 1984 5;GE-22(3):256–263. Available from: http://ieeexplore.ieee.org/lpdocs/epic03/wrapper.htm?arnumber=4157507. 10.1109/TGRS.1984.350619

[55] Crist E. A TM Tasseled Cap Equivalent Transformation for Reflectance Factor Data. Remote Sensing of Environment. 1985;306:301–306. Available from: http://www.sciencedirect.com/science/article/pii/0034425785901026. 10.1016/0034-4257(85)90102-6

[56] Gómez C, White JC, Wulder Ma, Alejandro P. Historical forest biomass dynamics modelled with Landsat spectral trajectories. ISPRS Journal of Photogrammetry and Remote Sensing. 2014 7;93:14–28. Available from: http://linkinghub.elsevier.com/retrieve/pii/S0924271614000719. 10.1016/j.isprsjprs.2014.03.008

[57] Ahmed OS, Franklin SE, Wulder Ma. Interpretation of forest disturbance using a time series of Landsat imagery and canopy structure from airborne lidar. Canadian Journal of Remote Sensing. 2014 6;39(6):521–542. Available from: http://www.tandfonline.com/doi/abs/10.5589/m14-004. 10.5589/m14-004

[58] Huber PJ. Robust Estimation of a Location Parameter. The Annals of Mathematical Statistics. 1964;35(1):73–101. 10.1214/aoms/1177703732

[59] Broich M, Hansen MC, Potapov P, Adusei B, Lindquist E, Stehman SV. Time-series analysis of multi-resolution optical imagery for quantifying forest cover loss in Sumatra and Kalimantan, Indonesia. International Journal of Applied Earth Observation and Geoinformation. 2011 4;13(2):277–291. Available from: http://linkinghub.elsevier.com/retrieve/pii/S0303243410001340. 10.1016/j.jag.2010.11.004

[60] Bai J, Perron P. Computation and analysis of multiple structural change models. Journal of Applied Econometrics. 2003 1;18(1):1–22. Available from: http://doi.wiley.com/10.1002/jae.659. 10.1002/jae.659

[61] de Jong R, Verbesselt J, Zeileis A, Schaepman M. Shifts in Global Vegetation Activity Trends. Remote Sensing. 2013 3;5(3):1117–1133. Available from: http://www.mdpi.com/2072-4292/5/3/1117/. 10.3390/rs5031117

[62] Breiman L. Random forests. Machine learning. 2001;p. 5–32. 10.1023/A:1010933404324

[63] Strobl C, Boulesteix AL, Kneib T, Augustin T, Zeileis A. Conditional variable importance for random forests. BMC bioinformatics. 2008;9:307 10.1186/1471-2105-9-307 18620558 PMC2491635

[64] Bellfield H, Sabogal D, Goodman L, Leggett M. Case Study Report: Community-Based Monitoring Systems for REDD+ in Guyana. Forests. 2015;6(1):133–156. Available from: http://www.mdpi.com/1999-4907/6/1/133/. 10.3390/f6010133

[65] FAO. Changes in shifting cultivation in Africa. FAO forestry paper 50. Forestry department.; 1984.

[66] Molinario G, Hansen MC, Potapov PV. Forest cover dynamics of shifting cultivation in the Democratic Republic of Congo: a remote sensing-based assessment for 2000–2010. Environmental Research Letters. 2015;10(9):094009 Available from: http://stacks.iop.org/1748-9326/10/i=9/a=094009?key=crossref.255fa2c946c3df8fed3e20ccd9dd3612. 10.1088/1748-9326/10/9/094009

[67] Gonsamo A, D’odorico P, Pellikka P. Measuring fractional forest canopy element cover and openness—definitions and methodologies revisited. Oikos. 2013 9;122(9):1283–1291. Available from: http://doi.wiley.com/10.1111/j.1600-0706.2013.00369.x. 10.1111/j.1600-0706.2013.00369.x

[68] Confalonieri R, Foi M, Casa R, Aquaro S, Tona E, Peterle M, et al Development of an app for estimating leaf area index using a smartphone. Trueness and precision determination and comparison with other indirect methods. Computers and Electronics in Agriculture. 2013;96:67–74. Available from: 10.1016/j.compag.2013.04.019. 10.1016/j.compag.2013.04.019

[69] Schnitzer SA, Bongers F. Increasing liana abundance and biomass in tropical forests: Emerging patterns and putative mechanisms. Ecology Letters. 2011;14(4):397–406. Cited By 117. Available from: http://www.scopus.com/inward/record.url?eid=2-s2.0-79952504440&partnerID=40&md5=f912dcd8681b8d1534e8489795b4d5df. 10.1111/j.1461-0248.2011.01590.x 21314879

[70] Schnitzer SA, Bongers F, Wright SJ. Community and ecosystem ramifications of increasing lianas in neotropical forests. Plant Signaling and Behavior. 2011;6(4):598–600. Cited By 13. Available from: http://www.scopus.com/inward/record.url?eid=2-s2.0-79955578791&partnerID=40&md5=5b0e77dfd34d0b3770c4a0f0113af0c1. 10.4161/psb.6.4.15373 21494089 PMC3142402

[71] Senbeta F, Schmitt C, Denich M, Demissew S, Velk PLG, Preisinger H, et al The diversity and distribution of lianas in the Afromontane rain forests of Ethiopia. Diversity and Distributions. 2005 7;11(5):443–452. Available from: http://blackwell-synergy.com/doi/abs/10.1111/j.1366-9516.2005.00180.x. 10.1111/j.1366-9516.2005.00180.x

[72] Morris RJ. Anthropogenic impacts on tropical forest biodiversity: A network structure and ecosystem functioning perspective. Philosophical Transactions of the Royal Society B: Biological Sciences. 2010;365(1558):3709–3718. Cited By 0. Available from: http://www.scopus.com/inward/record.url?eid=2-s2.0-78650404299&partnerID=40&md5=d8e533f4d2df5529733b48c42bd31fa9. 10.1098/rstb.2010.0273 PMC2982004 20980318

[73] Irons JR, Dwyer JL, Barsi Ja. The next Landsat satellite: The Landsat Data Continuity Mission. Remote Sensing of Environment. 2012 7;122:11–21. Available from: http://linkinghub.elsevier.com/retrieve/pii/S0034425712000363. 10.1016/j.rse.2011.08.026

[74] Li P, Jiang L, Feng Z. Cross-comparison of vegetation indices derived from landsat-7 enhanced thematic mapper plus (ETM+) and landsat-8 operational land imager (OLI) sensors. Remote Sensing. 2013;6(1):310–329. 10.3390/rs6010310

[75] Reiche J, Verbesselt J, Hoekman D, Herold M. Fusing Landsat and SAR time series to detect deforestation in the tropics. Remote Sensing of Environment. 2015 1;156:276–293. Available from: http://linkinghub.elsevier.com/retrieve/pii/S0034425714003885. 10.1016/j.rse.2014.10.001

