# Supplementary figures and images for: Characterizing Forest Change Using Community-Based Monitoring Data and Landsat Time Series
Source: PLoS One. 2016 Mar 28;11(3):e0147121. doi: 10.1371/journal.pone.0147121 (PMC4809496; doi:10.1371/journal.pone.0147121)

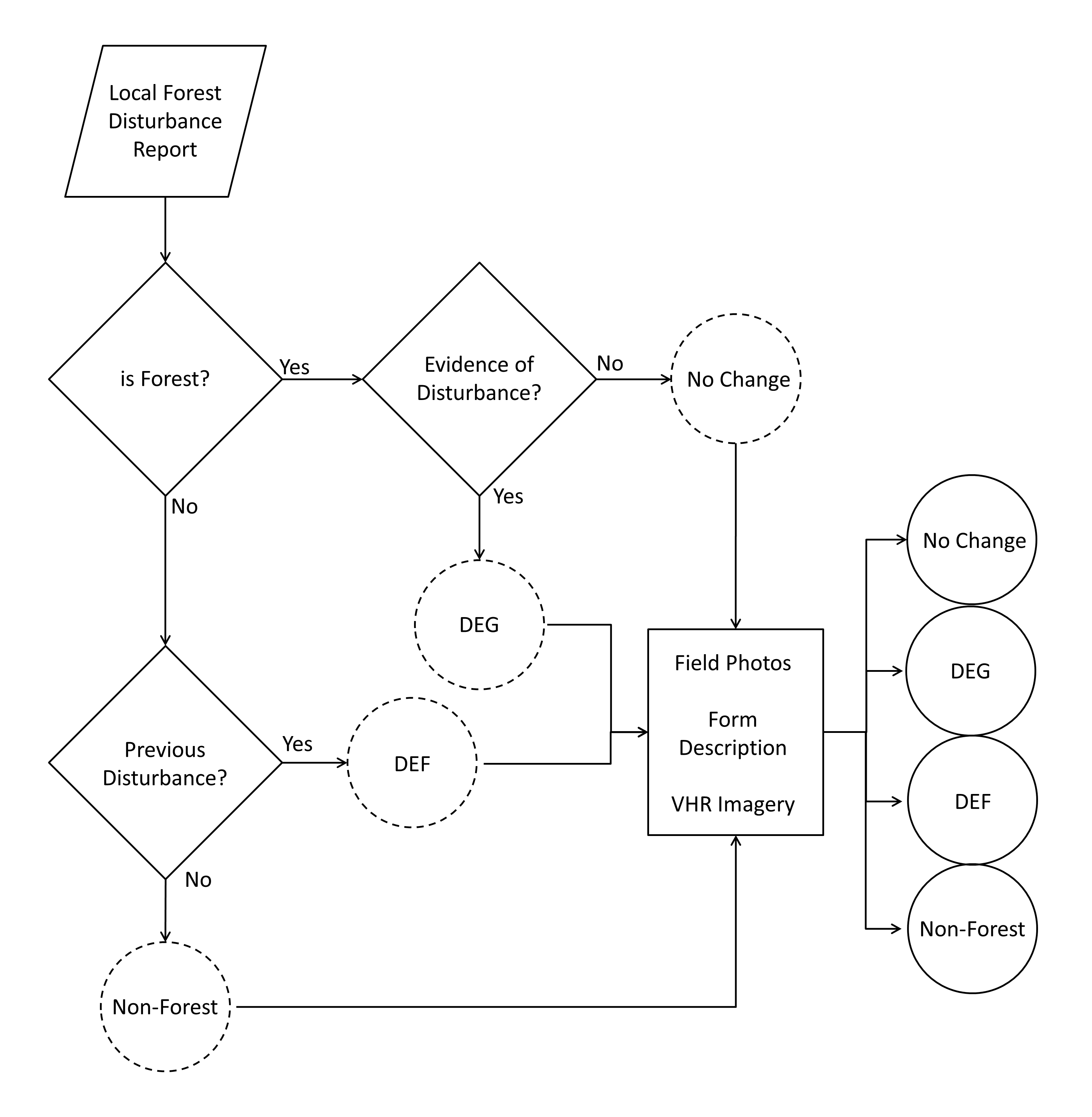

Supplement: S1 Fig — The primary class labels deforestation (DEF), degradation (DEG), no change (stable forest) or non-forest were assigned based on automatic interpretation of form attributes (circles with hatched outlines). Primary class labels were then verified using plot photos, plot descriptions and very high resolution (VHR) satellite imagery and final class labels (circles with solid outlines) were assigned. (TIFF) [file pone.0147121.s001.tiff]
