# Supplementary material for: Characterizing Forest Change Using Community-Based Monitoring Data and Landsat Time Series
Source: PLoS One. 2016 Mar 28;11(3):e0147121. doi: 10.1371/journal.pone.0147121 (PMC4809496; doi:10.1371/journal.pone.0147121)
Supplement: S1 Table — Coefficients used to transform reflectance bands from all Landsat sensors into tasseled cap indices. (PDF) [file pone.0147121.s002.pdf]

Table S1 - Tasseled Cap coefficients for surface reflectance data

Coefficients used to transform reflectance bands from all Landsat sensors into tasseled cap indices.

	B	G	R	NIR	SWIR1	SWIR2
TCB (b)	0.2043	0.4158	0.5524	0.5741	0.3124	0.2303
TCG (g)	-0.1603	0.2819	-0.4934	0.7940	-0.0002	-0.1446
TCW (w)	0.0315	0.2021	0.3102	0.1594	-0.6806	-0.6109
