# Supplementary material for: Characterizing Forest Change Using Community-Based Monitoring Data and Landsat Time Series
Source: PLoS One. 2016 Mar 28;11(3):e0147121. doi: 10.1371/journal.pone.0147121 (PMC4809496; doi:10.1371/journal.pone.0147121)
Supplement: S2 Table — Random forest scores based on overall accuracies and class accuracies for deforestation (DEF), degradation (DEG), no-change (NOCH). Spectral bands are shown from highest to lowest overall importance scores. (PDF) [file pone.0147121.s003.pdf]

Table S2 - Random forest importance scores

Random forest scores based on overall accuracies and class accuracies for deforestation (DEF), degradation (DEG), no-change (NOCH). Spectral bands are shown from highest to lowest overall importance scores.

Band	Overall	DEF	DEG	NOCH
SWIR2	0.986	1	0.795	0.769
TCW	0.937	0.655	0.892	0.951
NDMI	0.818	0.737	0.652	0.662
TCA	0.747	0.794	0.764	0.266
R	0.689	0.923	0.305	0.644
NDVI	0.645	0.424	0.723	0.482
G	0.529	0.617	0.059	0.967
NBR	0.475	0.569	0.348	0.432
SWIR1	0.401	0.491	0.027	0.845
TCB	0.237	0.283	0.281	0.159
B	0.219	0.006	0.521	0.282
NBR2	0.163	0.093	0.171	0.466
TCG	0.154	0.144	0.957	0.077
NIR	0.001	0.264	0.503	0
